# Lactate Enhances CD8^+^ T Cell Cytotoxicity Through H3K9la Upregulation to Drive Vitiligo Pathogenesis

**DOI:** 10.3390/ijms27093795

**Published:** 2026-04-24

**Authors:** Hang Yin, Yufei Xu, Luling Huang, Yuxuan Qian, Qing Zhu, Jianru Chen

**Affiliations:** 1 Department of Dermatology, Xijing Hospital, Fourth Military Medical University, Xi’an 710032, China; yinhang0201@163.com (H.Y.); xyf1041632550@163.com (Y.X.); huangluling0908@163.com (L.H.); yuxuanqian1@outlook.com (Y.Q.); zhuqing110300@163.com (Q.Z.); 2 National Key Laboratory of Immunity and Inflammation, Institute of Immunology, Naval Medical University, Shanghai 200433, China

**Keywords:** vitiligo, CD8^+^ T cells, autoimmunity, lactylation

## Abstract

Vitiligo is characterized by epidermal melanocyte destruction, with autoreactive CD8^+^ T cells playing a central pathogenic role, yet the mechanisms driving their hyperactivation remain unclear. Lactate has emerged as a key immunometabolite that functions as both a signaling molecule and an epigenetic modulator via protein lactylation. Nevertheless, the role of lactate in vitiligo pathogenesis has not been explored. Here, we report that serum lactate levels are significantly elevated in vitiligo patients and correlate positively with disease activity. In a mouse model, lactate administration accelerated vitiligo progression, accompanied by increased CD8^+^ T cell infiltration and melanocyte destruction in lesional skin. In vitro, lactate enhanced CD8^+^ T cell effector molecule expression (granzyme B, perforin, IFN-γ, CD107a) and cytotoxic function. Mechanistically, lactate increased global protein lactylation in CD8^+^ T cells, with marked enrichment at histone H3 lysine 9 (H3K9). H3K9 lactylation (H3K9la) was associated with enhanced chromatin accessibility and transcriptional activation of effector genes, as revealed by RNA sequencing and CUT&Tag analyses. Pharmacological inhibition of lactate production or lactylation abrogated these effects. Collectively, our findings identify lactate as a critical driver of CD8^+^ T cell pathogenicity in vitiligo through H3K9la-mediated epigenetic reprogramming, highlighting lactate metabolism and lactylation as potential therapeutic targets.

## 1. Introduction

Vitiligo, a common acquired depigmenting disorder affecting approximately 0.36% of the global population [1], imposes a substantial psychosocial burden due to the conspicuous lesions on exposed areas like the face and hands [2,3]. Accumulating evidence has firmly established that autoreactive CD8^+^ T cells targeting melanocytes play a central role in vitiligo pathogenesis, resulting in characteristic white macules and patches [4,5]. Nevertheless, the precise mechanisms governing the aberrant activation and persistence of these CD8^+^ T cells within the skin microenvironment remain incompletely elucidated.

Recent advances in immunometabolism have provided a new lens for understanding T cell dysfunction in autoimmunity [6,7,8]. While multiple metabolites, such as succinate, itaconate, and α-ketoglutarate, have been implicated in immune regulation, lactate possesses unique features that make it particularly relevant to CD8^+^ T cell biology in autoimmune settings. In inflammatory states, active T cells undergo a profound metabolic shift from oxidative phosphorylation to aerobic glycolysis, leading to substantial accumulation of lactate [9,10,11]. The resulting fluctuation in lactate concentration far exceeds that of other metabolites. Moreover, emerging evidence suggests that lactate plays a particular role in regulating CD8^+^ T cell stemness, exhaustion, and effector function through multiple mechanisms, including receptor engagement (e.g., GPR81) and transcriptional regulation [12,13,14,15,16]. The immunomodulatory role of lactate has been reported in various autoimmune diseases. For example, in rheumatoid arthritis, lactate accumulates in the inflamed synovium and is taken up by CD4^+^ T cells via SLC5A12, driving metabolic reprogramming and pro-inflammatory signals (e.g., PKM2/STAT3 and RORγt) that shift the Th17/Treg balance toward a pathogenic Th17 phenotype [17,18]. Similarly, lactate has been implicated in the pathogenesis of Sjögren’s syndrome by promoting the retention of CD4^+^ T cells and upregulating the expression of pro-inflammatory cytokines such as IL-17 [19]. However, its specific role in other diseases remains controversial [20,21]. For instance, in septic acute kidney injury, lactate upregulates PD-L1 and induces immunosuppression [22]. Furthermore, lactate suppresses the inflammatory responses of various immune cells in tumors, thereby promoting malignant tumor growth [12]. This controversy likely stems from a lack of mechanistic understanding at the cellular level. Notably, the specific role of lactate in vitiligo and the mechanisms by which lactate influences autoreactive CD8^+^ T cells remain largely unexplored. Further investigation is required to determine whether lactate influences the activation, proliferation, or cytotoxic activity of CD8^+^ T cells, and how this might disrupt immune homeostasis to promote melanocyte destruction.

The discovery of lysine lactylation (Kla) provides new insights into the molecular mechanisms by which lactate regulates CD8^+^ T cells. This post-translational modification occurs when lactate-derived moieties are covalently linked to lysine residues on histones [15], directly translating fluctuations in metabolic flux into transcriptional outputs. Notably, lactylation has been implicated in determining CD8^+^ T cell differentiation fate and functional subset characteristics, suggesting that lactylation constitutes a “metabolic-epigenetic” regulatory axis capable of influencing T cell-mediated autoimmunity [23]. However, despite these insights into its role in general CD8^+^ T cell biology, whether and how lactate drives lactylation in autoreactive CD8^+^ T cells remains to be explored. Furthermore, it is unclear how this modification subsequently modulates their pathogenic functions in autoimmune diseases. Elucidating this lactate-lactylation-CD8^+^ T cell axis in the context of autoimmunity may uncover novel therapeutic targets for disorders such as vitiligo.

Given the pivotal role of CD8^+^ T cells in vitiligo and the emerging link between lactate and CD8^+^ T cell function, we hypothesized that lactate contributes to vitiligo pathogenesis via lactylation-mediated epigenetic reprogramming. To test this hypothesis, we first measured serum lactate levels in vitiligo patients and analyzed their correlation with disease activity. We then employed a mouse model of vitiligo to examine the in vivo effects of lactate on disease progression and CD8^+^ T cell infiltration. In vitro, we investigated whether lactate directly modulates CD8^+^ T cell effector function and tried to delineate the underlying mechanisms.

## 2. Results

### 2.1. Lactate Promotes the Progression of Vitiligo

**Figure 1:**
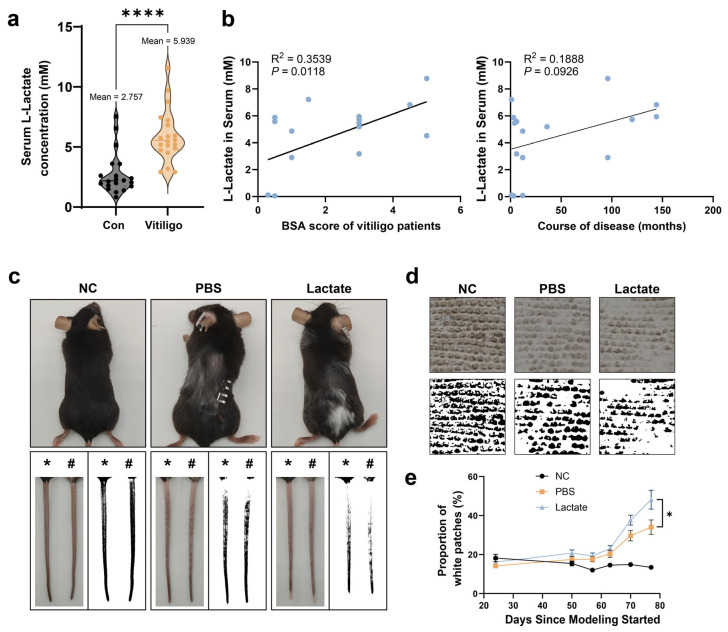
Lactate is involved in the pathogenesis of vitiligo. (**a**) Serum lactate concentrations in vitiligo patients and healthy controls were measured (n = 20). (**b**) Correlation analysis between serum lactate concentration and BSA score, as well as age, in vitiligo patients (n = 17). (**c**–**e**) Representative photographs (**c**) of whole tails (* dorsal, # ventral) and close-up views (**d**) of depigmented areas from mice in the normal control (NC), vitiligo model (PBS), and lactate-treated vitiligo model (Lactate) groups, along with quantitative analysis (**e**) of the depigmented area percentage. Data are presented as mean ± SEM. * *p* < 0.05, **** *p* < 0.0001 (unpaired two-tailed *t*-test for A; Pearson correlation coefficient test for B; one-way ANOVA with Dunnett’s test for F).

To investigate the potential role of lactate in vitiligo development and progression, we first analyzed serum L-lactate levels in a cohort comprising 20 active vitiligo patients (16 males, 4 females; mean age 26.30 ± 8.69 years) and 20 age- and sex-matched healthy controls (13 males, 7 females; mean age 27.95 ± 7.80 years. No statistically significant differences in age or gender distribution were observed between the two groups (*p* > 0.05, Table 1). Notably, serum L-lactate concentrations were significantly elevated in vitiligo patients compared to healthy controls (Figure 1a). Lactate levels were positively correlated with BSA scores but showed no significant association with disease duration (Figure 1b and Figure S1a). To further investigate the role of lactate in vitiligo pathogenesis, we employed a well-established mouse model of vitiligo induced by inoculation with B16F10 melanoma cells combined with regulatory T (Treg) cell depletion, as previously described [24] (Supplementary Figure S1b). Vitiligo mice received daily intraperitoneal injections of either lactate (500 mg/kg body weight) or PBS. Over five consecutive weeks, vitiligo mice progressively developed white hair on the back and evident depigmentation on both the ventral and dorsal tail, whereas no significant changes were observed in normal controls (Figure 1c,d). Notably, lactate-treated vitiligo mice exhibited faster disease progression than PBS-treated mice, as reflected by a higher percentage of tail depigmentation area at the same time points (Figure 1c–e). These results suggested that L-lactate might contribute to vitiligo pathogenesis and exacerbate disease progression.

### 2.2. Lactate Facilitates the Infiltration of Cytotoxic CD8^+^ T Cells in Vitiligo

**Figure 2:**
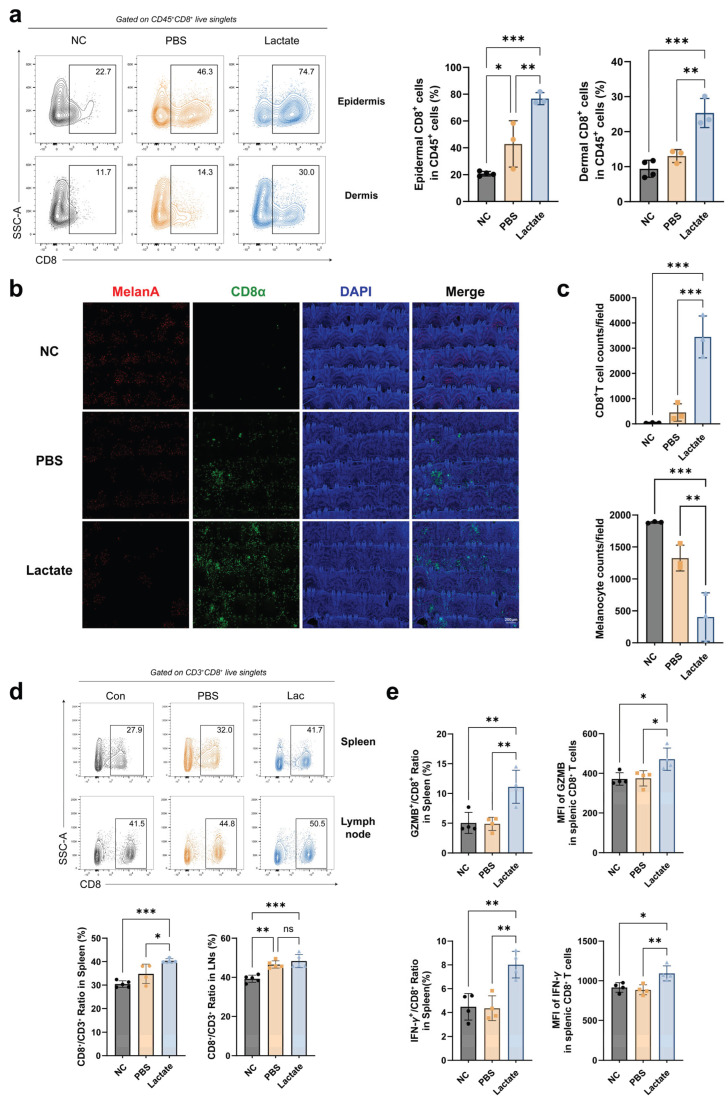
Lactate promotes CD8^+^ T cell infiltration in the vitiligo mouse model. (**a**) Flow cytometric analysis of the frequency of CD8^+^ T cells within the epidermis and dermis of mouse tails from the normal control (NC), vitiligo model (PBS), and lactate-treated groups. (**b**,**c**) Whole-mount immunofluorescence staining (**b**) of tail epidermis showing melanocytes (red) and infiltrating CD8^+^ T cells (green), with corresponding quantitative counts (**c**) for both cell types (n = 3). Nuclei were counterstained with DAPI (blue). (**d**) Representative flow cytometry plots and quantitative analysis of the frequency of CD8^+^ T cells within the spleen and lymph nodes of mice in the indicated groups. (**e**) Flow cytometric quantitative analysis of Granzyme B and IFN-γ expression in CD8^+^ T cells isolated from the spleens of mice in the indicated groups (n = 4). Data are presented as mean ± SEM. ns: *p* > 0.05, * *p* < 0.05, ** *p* < 0.01, *** *p* < 0.001 (one-way ANOVA with Dunnett’s test).

To further elucidate the role of lactate in vitiligo pathogenesis, we examined the distribution of epidermal melanocytes and CD8^+^ T cells in mouse tail skin using flow cytometry and whole-mount immunostaining. Flow cytometric analysis revealed that the proportion of CD8^+^ T cells in the tail epidermis was significantly increased in vitiligo mice compared to normal controls (Figure 2a and Figure S2a,b). Furthermore, the percentages of CD8^+^ T cells in both the epidermis and dermis were significantly higher in lactate-treated vitiligo mice than in PBS-treated mice (Figure 2a). Consistent with this, whole-mount immunostaining analysis showed substantial CD8^+^ T cell infiltration and marked melanocyte destruction in the tail epidermis of PBS-treated vitiligo mice relative to normal controls (Figure 2b). Notably, lactate-treated vitiligo mice exhibited significantly higher numbers of infiltrating CD8^+^ T cells and more pronounced melanocyte loss than PBS-treated mice, further supporting the role of lactate in promoting CD8^+^ T cell accumulation and tissue destruction (Figure 2c). Additionally, lactate treatment significantly increased the proportion of CD8^+^ T cells within the CD3^+^ population in the spleen and enhanced the expression of effector molecules (granzyme B, IFN-γ) in splenic CD8^+^ T cells (Figure 2d,e and Figure S2c–e). These results demonstrated that lactate promotes the infiltration of effector CD8^+^ T cells in both the circulation and lesional skin of vitiligo mice.

### 2.3. Lactate Enhances the Effector Function of CD8^+^ T Cells

**Figure 3:**
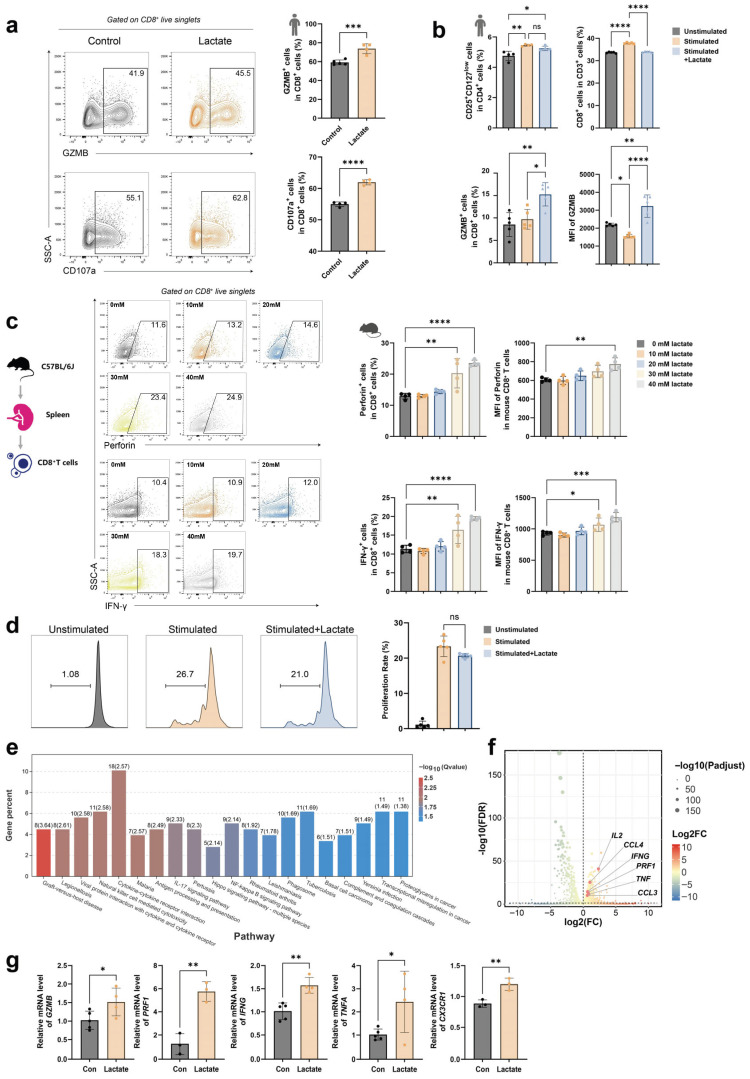
Lactate enhances the cytotoxic function of CD8^+^ T cells. (**a**,**b**) Representative flow cytometry plots (**a**) and quantitative analysis (**b**) of Granzyme B and CD107a expression in primary human CD8^+^ T cells from the Control and Lactate-treated groups (n = 3). (**c**) Representative flow cytometry plots and quantitative analysis of Perforin and IFN-γ expression in mouse splenic CD8^+^ T cells treated with control or varying concentrations of Lactate (10 mM, 20 mM, 30 mM, and 40 mM) (n = 3). (**d**) Representative flow cytometry histograms showing the proliferation of CFSE-labeled human CD8^+^ T cells following Lactate treatment, as assessed by CFSE dilution (n = 3). (**e**) KEGG pathway enrichment analysis of the significantly upregulated genes in human CD8^+^ T cells following Lactate treatment. (**f**) Volcano plot depicting DEGs in human CD8^+^ T cells from the Lactate-treated versus Control groups, as determined by RNA sequencing. (**g**) qPCR analysis of the mRNA expression levels of canonical effector genes in human CD8^+^ T cells from the Control and Lactate-treated groups (n = 3). Data are presented as mean ± SEM. ns: *p* > 0.05, * *p* < 0.05, ** *p* < 0.01, *** *p* < 0.001, **** *p* < 0.0001 (unpaired two-tailed *t*-test for A, F; one-way ANOVA with Dunnett’s test for B, C, and G).

Given that CD8^+^ T cell-mediated killing of melanocytes is central to vitiligo pathogenesis, we investigated the direct effects of L-lactate on CD8^+^ T cell effector function using in vitro assays. Based on cytotoxicity assays, the concentration of sodium lactate for in vitro treatment of CD8^+^ T cells was set at 40 mM (Supplementary Figure S3a). Treatment of primary human CD8^+^ T cells with sodium lactate similarly promoted the expression of granzyme B and CD107a (Figure 3a and Figure S3b). Specifically, lactate significantly increased granzyme B secretion by CD8^+^ T cells within PBMCs, while the proportion of Treg cells remained unchanged, suggesting that lactate modulates CD8^+^ T cell effector function directly rather than indirectly through Tregs (Figure 3b and Figure S3c). To exclude potential species-specific differences, these findings were validated in mouse CD8^+^ T cells, where lactate treatment similarly promoted effector function (Figure 3c and Figure S3d). Furthermore, lactate did not significantly affect CD8^+^ T cell proliferation, suggesting that its regulatory role in effector function occurs independently of proliferative expansion (Figure 3d). Transcriptome sequencing of lactate-treated CD8^+^ T cells revealed that the upregulated differentially expressed genes included multiple effector genes (e.g., *GZMB*, *IL2*, *IFNG*, *PRF1*, *TNF*, *CCL4*, and *CCL3*) and were significantly enriched in inflammation-related signaling pathways (Figure 3e,f and Figure S3e). QRT-PCR corroborated these findings (Figure 3g). Collectively, these results demonstrated that L-lactate directly upregulates effector-related gene expression, thereby enhancing the cytotoxic function of CD8^+^ T cells.

### 2.4. Endogenous and Exogenous Lactate Synergistically Promote CD8^+^ T Cell Effector Function

**Figure 4:**
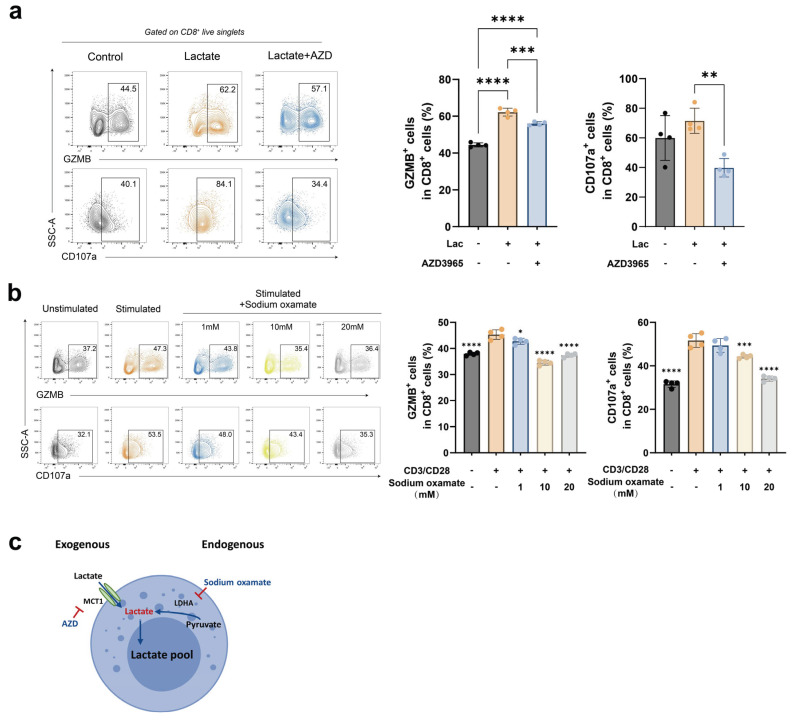
Endogenous and exogenous lactate synergistically enhance CD8^+^ T cell effector function. (**a**) Representative flow cytometry plots and quantitative analysis of Granzyme B and CD107a expression in human CD8^+^ T cells from the Control, Lactate-treated, and Lactate + AZD3965-treated groups (n = 4). (**b**) Representative flow cytometry plots and quantitative analysis of Granzyme B and IFN-γ expression in human CD8^+^ T cells from the Unstimulated, Stimulated, and Stimulated + Sodium oxamate-treated groups (n = 4). (**c**) Schematic diagram illustrating the sources of intracellular lactate in CD8^+^ T cells. Data are presented as mean ± SEM. * *p* < 0.05, ** *p* < 0.01, *** *p* < 0.001, **** *p* < 0.0001 (one-way ANOVA with Dunnett’s test for A, B).

Lactate in CD8^+^ T cells can originate from two sources: exogenous uptake and endogenous metabolism. Exogenous lactate is primarily transported into cells via monocarboxylate transporter 1 (MCT1) along its concentration gradient. Endogenous lactate is generated from pyruvate in a reaction catalyzed by lactate dehydrogenase A (LDHA). To determine whether both sources contribute to CD8^+^ T cell effector function, we used specific inhibitors to block the corresponding pathway. Blocking exogenous lactate transport via MCT1 with AZD3965 significantly reduced effector molecule expression in lactate-treated CD8^+^ T cells (Figure 4a and Figure S4a). Similarly, inhibiting endogenous lactate production with oxamate also significantly decreased effector molecule expression (Figure 4b and Figure S4b). These results indicate that exogenous and endogenous lactate act synergistically to promote CD8^+^ T cell effector function (Supplementary Figure S4c). Thus, lactate from both sources contributed to a common intracellular lactate pool that drove the enhancement of CD8^+^ T cell effector functions (Figure 4c).

### 2.5. Lactate Exerts Pro-Inflammatory Effects by Enhancing H3K9 Lactylation

**Figure 5:**
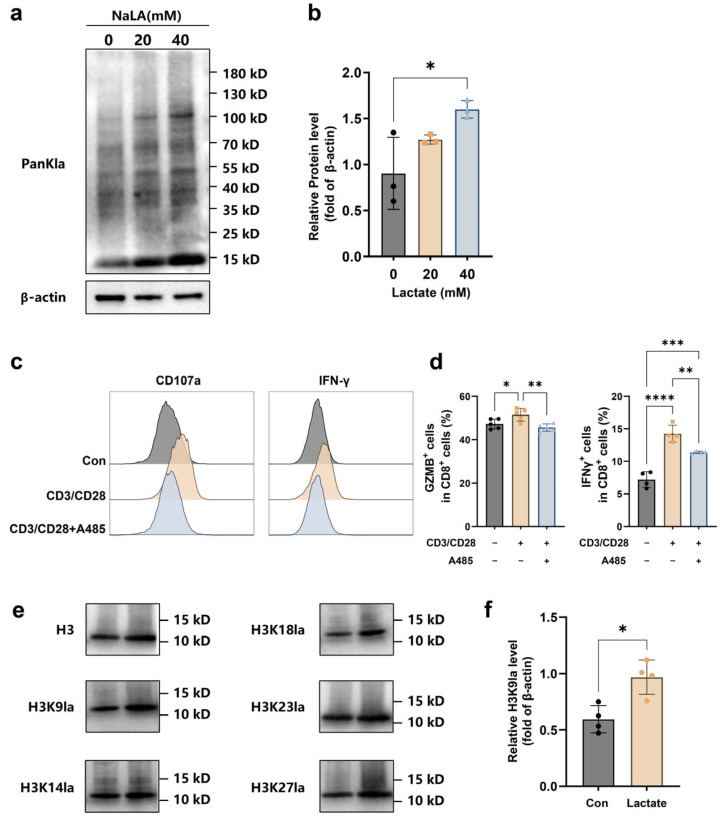
Lactate enhances CD8^+^ T cell cytotoxicity by increasing histone lactylation. (**a**) Western blot images and quantitative analysis of global protein lactylation levels in human CD8^+^ T cells from the Control group and groups treated with varying concentrations of Lactate (20 mM and 40 mM) (n = 3). (**b**) Western blot images of histone lactylation and quantitative analysis of H3K9la levels in human CD8^+^ T cells from the Control and Lactate-treated groups (n = 3). (**c**,**d**) Representative flow cytometry plots and quantitative analysis of Granzyme B and IFN-γ expression in CD8^+^ T cells from the Unstimulated, Stimulated, and Stimulated + A485-treated groups (n = 5). (**e**) Lactylation levels at various sites of histone H3 in human CD8^+^ T cells from the Control and Lactate-treated group. Total H3 blot was used as a loading control. (**f**) H3K9la levels in human CD8^+^ T cells from the Control and Lactate-treated groups (n = 4). Data are presented as mean ± SEM. * *p* < 0.05, ** *p* < 0.01, *** *p* < 0.001, **** *p* < 0.0001 (one-way ANOVA with Dunnett’s test for A and C; unpaired two-tailed *t*-test for B).

Although our results demonstrated that lactate upregulates effector-related gene expression and promotes CD8^+^ T cell cytotoxic function, the underlying mechanisms remained unclear. Given that lactate serves as a key substrate for protein lactylation, we investigated the effect of lactate on global protein lactylation levels in CD8^+^ T cells. Sodium lactate treatment significantly increased overall protein lactylation (Figure 5a,b). Crucially, blocking lactylation with A485, an inhibitor of lactylation, significantly decreased effector molecule expression in CD8^+^ T cells (Figure 5c,d). These findings suggested that blocking this specific modification impairs CD8^+^ T cell effector function. Lactylation is known to occur predominantly on specific histone sites, leading to epigenetic changes that modulate gene expression. Analysis of lactylation at specific histone modification sites revealed that lactate treatment specifically increased lactylation at the H3K9 site (H3K9la) in CD8^+^ T cells (Figure 5e,f). These findings collectively suggested that lactate promotes effector gene expression, potentially by increasing histone lactylation, particularly at the H3K9 site, in CD8^+^ T cells.

### 2.6. H3K9la Enhances the Cytotoxic Function of CD8^+^ T Cells by Promoting the Expression of Downstream Effector Genes

**Figure 6:**
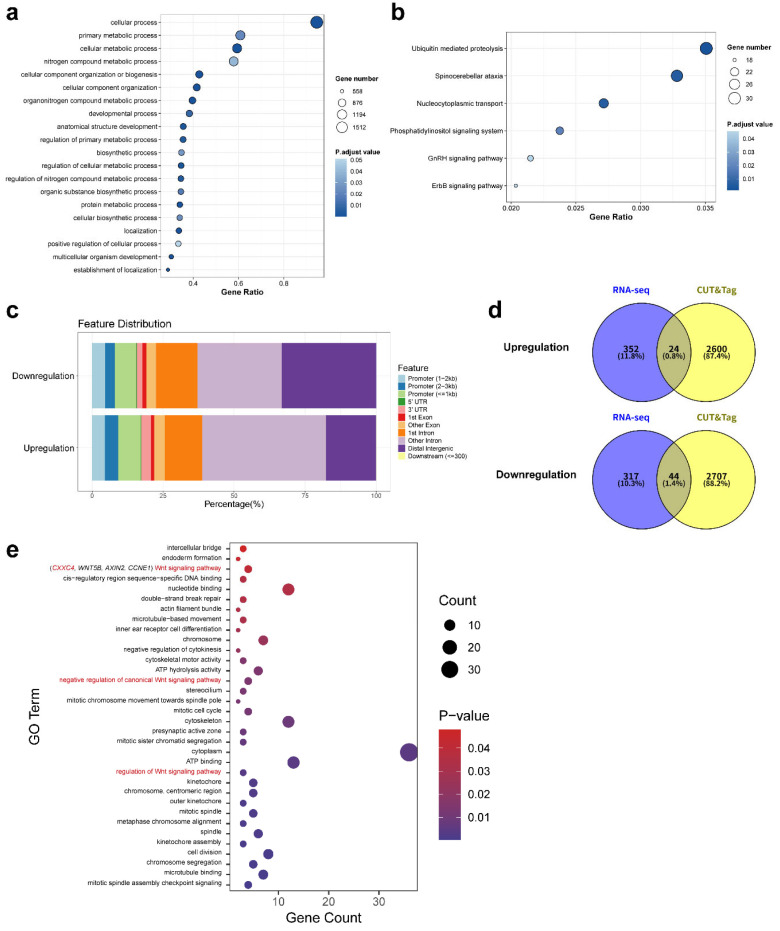
H3K9la Enhances the Cytotoxic Function of CD8^+^ T Cells by Promoting the Expression of Downstream Effector Genes. (**a**) Gene Ontology (GO) enrichment analysis of genes associated with H3K9la enrichment in human CD8^+^ T cells. (**b**) Kyoto Encyclopedia of Genes and Genomes (KEGG) pathway enrichment analysis of genes associated with H3K9la enrichment in human CD8^+^ T cells. (**c**) Bar chart showing the functional annotation distribution of differential H3K9la peaks between the Lactate-treated and Control groups. (**d**) Venn diagram depicting the overlap between differentially expressed genes in human CD8^+^ T cells identified by RNA sequencing and genes associated with differential H3K9la enrichment in human CD8^+^ T cells identified by CUT&Tag sequencing. (**e**) GO enrichment analysis of overlapping DEGs. Data are presented as mean ± SEM.

To identify the genes potentially regulated by H3K9la, we performed CUT&Tag sequencing to map H3K9la-associated genes and examine their distribution across functional genomic regions. GO and KEGG enrichment analyses of genes associated with H3K9la peaks revealed their broad involvement in metabolic activation, protein synthesis, and signal transduction, providing potential mechanistic insights into how H3K9la promotes CD8^+^ T cell effector function (Figure 6a,b). Notably, the proportion of genes with H3K9la enrichment at promoter regions was increased among upregulated genes, supporting a role for H3K9la in facilitating gene expression (Figure 6c). Integrating these data with our previous transcriptomic analysis, we performed an overlap analysis between the H3K9la-associated differentially expressed genes (DEGs) and the lactate-regulated genes. (Figure 6d). Then, we performed GO enrichment analyses and identified genes involved in T cell function-related pathways that also exhibited H3K9la enrichment at their promoters (Figure 6e). This led to the identification of *CXXC4* as a candidate gene directly regulated by H3K9la in CD8^+^ T cells.

## 3. Discussion

This study identifies lactate as a driver of CD8^+^ T cell pathogenicity in vitiligo through H3K9la-mediated epigenetic reprogramming. We found that serum lactate levels are significantly elevated in patients with vitiligo and correlate with disease activity. In a mouse model, lactate administration accelerated disease progression, accompanied by increased CD8^+^ T cell infiltration and melanocyte destruction. Mechanistically, lactate enhanced CD8^+^ T cell effector function by upregulating H3K9 lactylation, thereby epigenetically modulating effector gene expression. These findings position lactate not merely as a metabolic byproduct but as a key signaling molecule linking metabolism to immunity in vitiligo.

The elevated serum lactate levels in vitiligo patients may originate from two sources: endogenous lactate produced by locally infiltrating immune cells undergoing aerobic glycolysis and exogenous lactate resulting from systemic metabolic changes (e.g., obesity, insulin resistance, stress) [25,26]. The multiplicity of intracellular lactate sources provides a novel perspective for clinical management. Current treatments for vitiligo mainly focus on local immunosuppression within the affected skin [27]. Our findings suggest that for patients with existing systemic metabolic issues, combining lifestyle changes or metabolism-targeting drugs with standard topical therapy might lower systemic lactate levels, indirectly reducing the pro-inflammatory metabolic environment in the skin, which could improve treatment results and lower the chance of relapse. However, this study did not quantify the relative contributions of endogenous versus exogenous lactate in influencing CD8^+^ T cell activity, an essential area for future research.

This study also reveals a significant elevation in serum lactate levels among patients with vitiligo, which correlates with disease progression, suggesting its potential as a biomarker for early diagnosis. Clinically, vitiligo is often diagnosed only after distinct skin depigmentation has developed, and achieving repigmentation remains therapeutically challenging. Consequently, identifying biomarkers capable of detecting vitiligo prior to the formation of visible lesions may represent a critical step toward disease prevention. The combined assessment of serum lactate and H3K9 lactylation could serve as a dynamic indicator and a biomarker of disease diagnosis. However, measuring serum lactate levels alone may not serve as a simple, direct, and sufficiently reliable routine clinical test. Therefore, further studies are required to develop early diagnostic markers for vitiligo, such as exploring histone lactylation and lactate-related metabolic pathways.

Notably, our findings demonstrate that lactate plays a key pro-inflammatory role in the pathogenesis of vitiligo. Similarly, in autoimmune diseases such as rheumatoid arthritis, lactate has been shown to promote IL-17 production by CD4^+^ T cells, exacerbating inflammation [12,17,18,28]. However, in the tumor microenvironment, lactate generally exerts immunosuppressive effects by enhancing Treg function and suppressing CD8^+^ T cell proliferation and effector activity, thereby facilitating immune evasion [12,14]. These findings suggest that lactate, a common and widely distributed immunometabolite, exerts complex regulatory effects on systemic metabolism and the immune system. Under pathological conditions, the specific biological outcome of lactate is dictated by the specific disease type, microenvironmental cues, and the identity of the target cells, ultimately resulting in either pro- or anti-inflammatory outcomes [12]. For this functional dichotomy, differences in microenvironmental pH can serve as one explanation [14]. In the tumor microenvironment, lactate accumulation driven by the Warburg effect is accompanied by a sharp decrease in pH [14]. In contrast, the pH in vitiligo lesions remains neutral, similar to normal skin, and may even be slightly elevated in some inflamed lesions [29,30]. Indeed, one study showed that lactate suppresses CD8^+^ T cell cytotoxicity under acidic conditions, an effect that was reversed upon neutralization of the extracellular pH [20]. Therefore, the neutral microenvironment enables lactate to exert pro-inflammatory effects in vitiligo, distinct from its immunosuppressive role in tumors. Additionally, metabolic competition between non-proliferative cells (e.g., melanocytes) and CD8^+^ T cells is less intense in vitiligo, positioning lactate primarily as a byproduct and signal of immune cell activation. These observations underscore the context-dependent nature of lactate immunomodulation and suggest that therapeutic strategies targeting lactate metabolism must account for disease-specific microenvironmental features.

Beyond the critical influence of pH, lactate exerts differential effects depending on the type of inflammation. In acute inflammation, lactate primarily plays an immunosuppressive role. Studies have shown that lactate inhibits the production of pro-inflammatory cytokines (e.g., TNF-α, IL-6) and suppresses the activation of inflammatory signaling pathways such as NF-κB in LPS-activated macrophages, thereby exerting anti-inflammatory effects. In contrast, lactate levels are frequently elevated in chronic inflammation, where it promotes local inflammatory progression through multiple mechanisms. For instance, lactate downregulates HK1/PKM2 in T cells, impairing energy supply and migration, which causes T cell retention and sustained chronic inflammation [17]. Lactate also upregulates SLC5A12 in CD4^+^ T cells, promoting IL-17 production via PKM/STAT3 [28]. In chronic inflammatory conditions such as autoimmune diseases, elevated lactate levels may indirectly reflect active aerobic glycolysis in locally activated immune cells within the lesional skin. This altered metabolic profile may indicate a “Warburg-like” metabolic pattern in autoimmune diseases, including vitiligo, positioning lactate as a signal of local immunometabolic activity and linking lesional inflammation with systemic immunometabolism. Future studies may build on these observations to explore deeper connections between the immunological pathogenesis of vitiligo and systemic metabolism.

Lactate also exerts cell-type-specific immunomodulatory effects. Our study demonstrates that lactate enhances the cytotoxic function of CD8^+^ T cells. In other research, lactate limits the pro-inflammatory function of dendritic cells through HIF-1α-NDUFA4L2 [31]. Additionally, it induces M2 macrophage polarization, conferring an anti-inflammatory phenotype [12]. These divergent responses across immune cell lineages highlight that lactate is not a monolithic signal but rather a context-dependent modulator. In specific disease settings, the integration of these opposing effects forms a regulatory network that balances immune activation and homeostasis. Ultimately, the net outcome of lactate signaling depends on the composition and functional state of the local immune microenvironment. Thus, the immunomodulatory role of lactate must be interpreted within the specific cellular and disease context, with particular attention to the identity of the target immune cell and the nature of the inflammatory milieu.

A seemingly contradictory observation arises from early clinical studies, in which topical or intralesional lactic acid was reported to have therapeutic benefits in vitiligo [32,33]. However, several key differences in the experimental and clinical context reconcile this discrepancy. First, in the therapeutic studies, lactic acid was applied topically (15% cream or solution) or injected intralesionally (1% solution) at intermittent intervals (e.g., weekly or biweekly) [32,33]. This results in transient, high-concentration pulses of lactate, which likely act locally on keratinocytes and the epidermal barrier rather than systemically on CD8^+^ T cells [34]. In contrast, in our study, lactate was elevated systemically (serum) and persistently in the inflammatory microenvironment, leading to sustained epigenetic reprogramming of autoreactive CD8^+^ T cells. Second, the pH microenvironment plays a decisive role. In the therapeutic studies, the applied lactic acid solutions were formulated at acidic pH (e.g., pH 6.84 for 1% injectable solution), which may enhance skin penetration and local irritation but does not reflect the neutral pH of the vitiligo lesional microenvironment [32,35]. Moreover, similar to the tumor microenvironment, lactate under acidic conditions tends to promote the immunosuppressive function of Treg cells [14]. In contrast, our in vitro experiments were performed at neutral pH, mimicking the physiological condition of inflamed skin in vitiligo [30]. Under neutral conditions, lactate is not protonated and can be taken up by CD8^+^ T cells via MCT1 transporters to drive H3K9 lactylation and effector gene expression. Thus, lactate can be either pathogenic or therapeutic depending on whether it arises from sustained endogenous metabolism or is delivered exogenously for transient topical treatment. Future studies need to explore disease-tailored strategies to therapeutically reprogram lactate signaling without disrupting homeostatic immune functions.

Histone lactylation, a pivotal epigenetic modification, offers a molecular basis for understanding how metabolic signals are converted into lasting transcriptional programs. Here, we found that lactate treatment significantly increased H3K9 lactylation in CD8^+^ T cells, which was associated with the activation of effector gene transcription (e.g., *GZMB*, *IFNG*). The H3K9 residue acts as a key epigenetic hub, capable of undergoing both repressive (e.g., H3K9me3) and activating (e.g., H3K9la, H3K9ac) modifications [36,37,38]. H3K9me3 can directly repress gene transcription by either recruiting or activating downstream effector factors (such as LDL2 in Arabidopsis) to remove activating histone marks (e.g., H3K4me1). Additionally, it can form a self-reinforcing heterochromatin loop with other modifications like DNA methylation, thereby stably maintaining gene silencing. In CD8^+^ T cells, the enrichment of H3K9la may competitively inhibit methylation at the same histone residue. This antagonistic effect potentially reduces the deposition of the repressive mark H3K9me, thereby alleviating transcriptional repression at pro-inflammatory gene loci and promoting gene expression. Furthermore, studies have shown that H3K9la can cooperate with activating mark H3K27ac to enhance gene transcription, which may represent one of the mechanisms through which lactylation drives the pro-inflammatory functions of the CD8^+^ T cell [39]. This “metabolic–epigenetic” regulatory pathway allows CD8^+^ T cells to rapidly sense and respond to metabolic changes in their environment, playing a key role in vitiligo pathogenesis.

From a translational perspective, this study highlights several potential therapeutic targets for vitiligo. One approach involves intervening in lactate production or transport. For instance, LDHA inhibitors such as *stiripentol* or MCT1 inhibitors like AZD3965 could reduce intracellular lactate levels, which consequently suppresses lactylation [40,41]. Nonetheless, systemic inhibition of lactate metabolism may disrupt energy homeostasis and signaling in normal cells. Alternatively, direct modulation of histone lactylation could be achieved through small molecules targeting lactylation-associated enzymes, such as the HDAC inhibitor *vorinostat* [42,43]. However, epigenetic interventions of this nature currently lack specificity for distinct histone residues or genomic loci. Future efforts may integrate genomic sequencing with protein editing technologies to develop targeted therapies that precisely modulate H3K9 lactylation in CD8^+^ T cells.

Although this study establishes a direct link between lactate and CD8^+^ T cell effector function, several unanswered questions highlight the complexity of the vitiligo lesional microenvironment. First, while we focused on the direct effects of lactate on CD8^+^ T cells, the vitiligo lesional microenvironment contains diverse immune cell populations (e.g., CD4^+^ T cells, macrophages, dendritic cells). The potential regulatory effects of lactate on these cell types and their crosstalk with CD8^+^ T cell function remain to be elucidated. Research on tumors has demonstrated that lactate suppresses dendritic cell maturation and induces M2 macrophage polarization [31,44,45]; in the context of vitiligo, such effects might indirectly modulate disease progression by influencing antigen presentation or local immune homeostasis. Second, histone modifications exist within a complex interactive network; the synergistic or antagonistic relationships between H3K9la and other modifications (e.g., H3K9ac, H3K27ac, H3K4me3) in regulating CD8^+^ T cell effector genes require systematic dissection using multi-omics approaches such as ChIP-seq. Additionally, the specific “writers” and “erasers” enzymes catalyzing H3K9 lactylation have yet to be definitively identified, limiting the development of precision interventions targeting this modification. Third, in this study, we established a mouse model of vitiligo by combining melanoma implantation with Treg depletion. This model recapitulates the key pathogenic process of vitiligo, specifically melanocyte-specific killing by CD8^+^ T cells. By reproducing the aberrant activation and lesional infiltration of pathogenic CD8^+^ T cells, as well as subsequent melanocyte destruction, it provides a convenient platform for studying the immunological mechanisms of vitiligo. However, it should be noted that in this model, CD8^+^ T cells are primarily induced by melanoma, and autoimmunity occurs only upon artificial Treg depletion [24]. Consequently, the model fails to recapitulate autoantigen exposure and immune tolerance breakdown, and thus cannot fully represent spontaneous vitiligo.

In conclusion, this study identifies the lactate-driven H3K9la-mediated enhancement of CD8^+^ T cell effector function as a pivotal mechanism in vitiligo pathogenesis, positioning lactate not merely as a metabolic byproduct but as an epigenetic modulator. These findings not only advance our understanding of vitiligo immunopathogenesis but also provide a novel therapeutic avenue for other autoimmune diseases characterized by intricate metabolic-immune crosstalk: targeting metabolic–epigenetic modifications. Future studies should further dissect the fine regulatory networks governing lactylation, evaluate the therapeutic potential and safety of targeting this pathway, and explore feasible paths for clinical translation.

## 4. Materials and Methods

### 4.1. Patients and Samples

Peripheral blood samples were collected from patients given a diagnosis of vitiligo according to clinical and histologic manifestations in the Department of Dermatology, Xijing Hospital of the Fourth Military Medical University. All patients included in the study displayed initiation of depigmentation within 3 months and had not received any systemic or topical therapy for at least 3 months before sample collection. Age- and sex-matched healthy controls were recruited from the Xijing Hospital Physical Examination Center during the same period. In this study, blood samples were collected from 20 patients with vitiligo and 20 healthy controls. Epidemiological data of the vitiligo patients, including sex, age, disease duration, and BSA score, were obtained through questionnaires completed during outpatient visits (see Table 1).

Venous blood from all participants was collected into EDTA anticoagulant tubes. Plasma was separated from 4 mL of whole blood by centrifugation and stored at −80 °C for subsequent analysis. Peripheral blood mononuclear cells (PBMCs) were isolated by density gradient centrifugation from the remaining blood for immediate use in functional assays. Briefly, blood was carefully layered onto an equal volume of human peripheral blood lymphocyte separation medium. Following centrifugation (2000 rpm, 20 min, 20 °C) with controlled acceleration and deceleration, the buffy coat layer containing PBMCs was collected. Cells were washed with PBS, and residual red blood cells were lysed using RBC lysis buffer. The isolated PBMCs were then resuspended in complete RPMI 1640 medium and cultured at 37 °C in a 5% CO_2_ incubator. CD8^+^ T cells were isolated from PBMCs using positive magnetic selection. PBMCs were incubated with CD8 MicroBeads in MACS Buffer for 15 min at 4 °C. The cell suspension was then applied to an LS column placed in a magnetic field. After washing, the column was removed from the magnet, and CD8^+^ T cells were eluted with MACS Buffer. The purified cells were collected by centrifugation, resuspended in complete RPMI 1640 medium, and cultured at 37 °C in a 5% CO_2_ incubator.

### 4.2. Cell Culture

Human PBMCs and CD8^+^ T cells, as well as mouse peripheral lymphocytes and splenic CD8^+^ T cells, were cultured in Modified RPMI 1640 medium supplemented with 10% fetal bovine serum (FBS) and 1% penicillin-streptomycin. The B16F10 melanoma cell line was cultured in high-glucose DMEM supplemented with 10% FBS and 1% penicillin-streptomycin.

### 4.3. Vitiligo Mouse Model and Treatment

All animal experiments were approved by the Animal Experiment Committee of Fourth Military Medical University. Mice were randomly assigned to three groups: a normal control group (NC, no manipulation), a PBS treatment group (PBS), and a sodium lactate treatment group (Lac, lactate). The vitiligo mouse model was established in 9-week-old C57BL/6 female mice by intradermal inoculation with mouse-derived B16F10 melanoma cells as previously described. The day of melanoma cell inoculation was designated as day 0 of model establishment. On days 4 and 10, a CD4-neutralizing antibody was injected intraperitoneally to accelerate the generation of autoreactive CD8^+^ T cells. On day 12, after surgical resection of the dorsal mass, the mouse vitiligo model was established. Successful modeling was generally assessed by whole-mount staining around day 35. Starting from day 13, the Lac group received daily intraperitoneal injections of sodium lactate (500 mg/kg body weight, dissolved in sterile PBS). The PBS received an equivalent volume of sterile PBS. Mice in the NC group were untreated healthy animals without model induction.

Mouse samples were collected at the experimental endpoint. Blood was obtained via retro-orbital bleeding under anesthesia. Mice were then euthanized by cervical dislocation. Tail skin (1–2 cm) was collected for immunofluorescence analysis. Spleens and bilateral inguinal lymph nodes were harvested, and single-cell suspensions were prepared by gently pressing the tissues through a 70-μm nylon mesh using a syringe plunger. Cells were washed with cold PBS and used for downstream applications.

### 4.4. Whole-Mount Immunostaining and Confocal Microscopy of Mouse Tail Epidermis

Tail skin was depliated, and the epidermis was separated from the dermis following incubation in 20 mM EDTA at 37 °C for 1.5–2 h. The epidermal sheets were fixed in 4% paraformaldehyde and then in cold methanol containing 0.3% H_2_O_2_. After blocking with BSA, the sheets were incubated overnight at 4 °C with primary antibodies: rabbit anti-mouse Melan-A (diluted 1:300) and rat anti-mouse CD8α (diluted 1:300). After washing, they were incubated for 2 h at room temperature with secondary antibodies: Cy3-conjugated donkey anti-rat IgG (diluted 1:400) and Alexa Fluor^®^ 647-conjugated donkey anti-rabbit IgG (diluted 1:400), along with DAPI (diluted 1:1000). Stained epidermal sheets were mounted and imaged using a confocal laser scanning microscope (Zeiss LSM 880, Thornwood, NY, USA). Z-stack images of the full epidermal thickness were acquired. For this study, the sample size of whole-mount immunostaining was 3 per group.

### 4.5. Flow Cytometry Analysis

Following the designated treatments, cell samples were harvested and stained with Zombie Dye UV to label dead cells for exclusion from downstream data analysis. For surface marker staining, single-cell suspensions were incubated with fluorochrome-conjugated antibodies in PBS for 30 min at room temperature in the dark. For intracellular staining, cells were first fixed and permeabilized using a fixation/permeabilization kit, then incubated with intracellular antibodies for 30 min at room temperature in the dark. After washing, cells were resuspended in PBS and analyzed on a flow cytometer. The flow cytometry antibodies used in the experiment include: APC anti-mouse CD8α (Biolegend, San Diego, CA, USA), FITC anti-mouse CD45 (Biolegend, San Diego, CA, USA), PerCP anti-mouse CD3 (Biolegend, San Diego, CA, USA), FITC anti-mouse CD3 (Biolegend, San Diego, CA, USA), Pacific Blue anti-mouse Perforin (Biolegend, San Diego, CA, USA), PE anti-mouse/human GZMB (Biolegend, San Diego, CA, USA), BV421 anti-mouse IFN-γ (Biolegend, San Diego, CA, USA), BV605 anti-mouse IFN-γ (Biolegend, San Diego, CA, USA), APC anti-human CD8α (Biolegend, San Diego, CA, USA), PE-eFluor610 anti-human CD107a (Invitrogen, Carlsbad, CA, USA), BV605 anti-human IFN-γ (Biolegend, San Diego, CA, USA), FITC anti-human CD3 (Biolegend, San Diego, CA, USA), PE anti-human CD127 (Biolegend, San Diego, CA, USA), PerCP anti-human CD25 (Biolegend, San Diego, CA, USA), and Alexa Fluor 700 anti-human CD4 (Biolegend, San Diego, CA, USA). Each flow cytometry experiment was performed with at least three biological replicates, and the exact sample sizes are indicated in the Results section.

### 4.6. L-Lactate Measurement

This experiment included a vitiligo group and a healthy control group, with 20 samples in each group. L-lactate concentrations in serum samples were determined using a Lactate Assay Kit (Beyotime, Shanghai, China), following the manufacturer’s instructions. Briefly, serum samples or L-lactate standards were added to a 96-well plate and brought to a volume of 50 μL with Lactate Assay Buffer. Then, 50 μL of WST-8 working solution was added to each well and incubated at 37 °C for 30 min, protected from light. Absorbance was measured at 450 nm. Sample concentrations were calculated based on a standard curve.

### 4.7. Quantitative Real-Time PCR (qRT-PCR)

To examine the expression of specific effector genes in CD8^+^ T cells, we quantified their mRNA levels by qPCR. Total RNA was extracted from human CD8^+^ T cells using an RNA isolation kit. RNA concentration was measured, and 1 μg of RNA was reverse-transcribed into cDNA using PrimeScript RT Master Mix (Takara Bio, Kusatsu, Japan). qRT-PCR was performed using TB Green Premix (Takara Bio, Kusatsu, Japan) on a real-time PCR system. Gene expression levels were calculated using the comparative cycle threshold (2^−ΔΔCt^) method. The sample size for the experimental and control groups ranged from 3 to 5, with three technical replicates per sample.

### 4.8. Western Blotting

Cells were lysed in RIPA buffer. Protein concentrations were determined using a BCA protein assay kit. Equal amounts of protein (50 μg) were separated by SDS-PAGE and transferred onto a PVDF membrane. Membranes were blocked and then incubated overnight at 4 °C with primary antibodies, followed by incubation with HRP-conjugated secondary antibodies (β-Actin) for 1 h at room temperature. Protein bands were visualized using an enhanced chemiluminescence (ECL) substrate and imaged. Densitometric analysis was performed using ImageJ software, version 1.54g. Each Western blot experiment was performed with three biological replicates. The following primary antibodies were used: Anti-L-Lactyl Lysine Rabbit mA (PTM BIO, Hangzhou, China), Anti-L-Lactyl-Histone H3 (Lys9) Rabbit mAb (PTM BIO, Hangzhou, China), Anti-L-Lactyl-Histone H3 (Lys14) Rabbit mAb (PTM BIO, Hangzhou, China), Anti-L-Lactyl-Histone H3 (Lys18) Rabbit mAb (PTM BIO, Hangzhou, China), Anti-L-Lactyl-Histone H3 (Lys23) Rabbit mAb (PTM BIO, Hangzhou, China), Anti-L-Lactyl-Histone H3 (Lys27) Rabbit mAb (PTM BIO, Hangzhou, China), Anti-Histone H3 Rabbit mAb (PTM BIO, Hangzhou, China).

### 4.9. RNA Sequencing and Data Analysis

Purified human CD8^+^ T cells from healthy controls were activated with CD3/CD28 and treated with or without 40 mM sodium lactate for 72 h. Three biological replicates were included in this experiment. Each replicate was divided equally between the treatment and control groups, giving a sample size of three per group. Total RNA was extracted, and library preparation and sequencing were performed by Genedenovo (Guangzhou, China). Differentially expressed genes were identified, followed by Gene Ontology (GO) and Kyoto Encyclopedia of Genes and Genomes (KEGG) pathway enrichment analyses.

### 4.10. CUT&Tag

Purified human CD8^+^ T cells from healthy controls were activated with CD3/CD28 and treated with or without 40 mM sodium lactate for 72 h. This experiment comprised three biological replicates. Each replicate was equally divided and allocated to the treatment and control groups, resulting in a sample size of three per group. Cells were then collected, cryopreserved, and submitted to PTM Bio (Hangzhou, China) for CUT&Tag sequencing to profile genome-wide histone modifications.

### 4.11. Statistical Analysis

Data are presented as mean ± SEM. Statistical significance for two-group comparisons was determined using a two-tailed Student’s *t*-test. For multiple group comparisons, one-way ANOVA was performed, followed by Tukey’s post hoc test to correct for family-wise error rate. A *p*-value < 0.05 was considered statistically significant. No a priori power analysis was performed; sample sizes were determined based on previous experience and relevant literature.

## Figures and Tables

**Table 1:** Demographic and clinical characteristics of the study population.

Variable	Vitiligo (n = 20)	Controls (n = 20)	*p* Value
Gender, n (%)			
Male	16 (80.0)	13 (65.0)	0.288
Female	4 (20.0)	7 (35.0)	
Age (years)	26.3 ± 8.69	27.95 ± 7.8	0.536
Disease duration (months), mean ± SD	43.5 ± 55.3		
BSA score	2.14 ± 1.66		

Data is shown as mean ± SD, and *p*-values are statistically significant (*p*  ≤  0.05). BSA, Body Surface Area. The Chi-square test is used for clinical manifestation variables.

## Data Availability

The data that support the findings of this study are available from the corresponding author upon reasonable request.

## Supplementary Materials

The following supporting information can be downloaded at: https://www.mdpi.com/article/10.3390/ijms27093795/s1.
