# Supplementary material for: Lactate Enhances CD8+ T Cell Cytotoxicity Through H3K9la Upregulation to Drive Vitiligo Pathogenesis
Source: Int J Mol Sci. 2026 Apr 24;27(9):3795. doi: 10.3390/ijms27093795 (PMC13164054; doi:10.3390/ijms27093795)
Supplement: Supplementary file 1 [file ijms-27-03795-s001.zip › ijms-4225324-supplementary.pdf]

Supplementary Materials

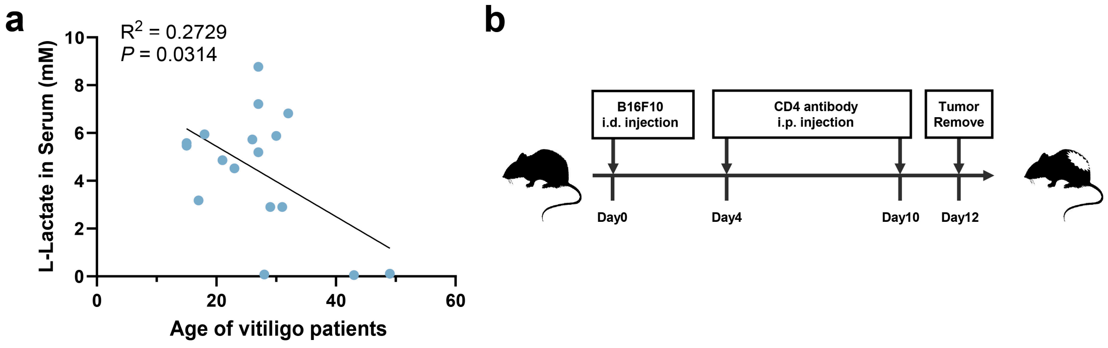

Supplementary Figure S1. Lactate is involved in the pathogenesis of vitiligo. (a) Correlation analysis between serum lactate concentration and disease duration in vitiligo patients. (Pearson correlation coefficient test) (b) Schematic diagram illustrating the establishment of the vitiligo mouse model.

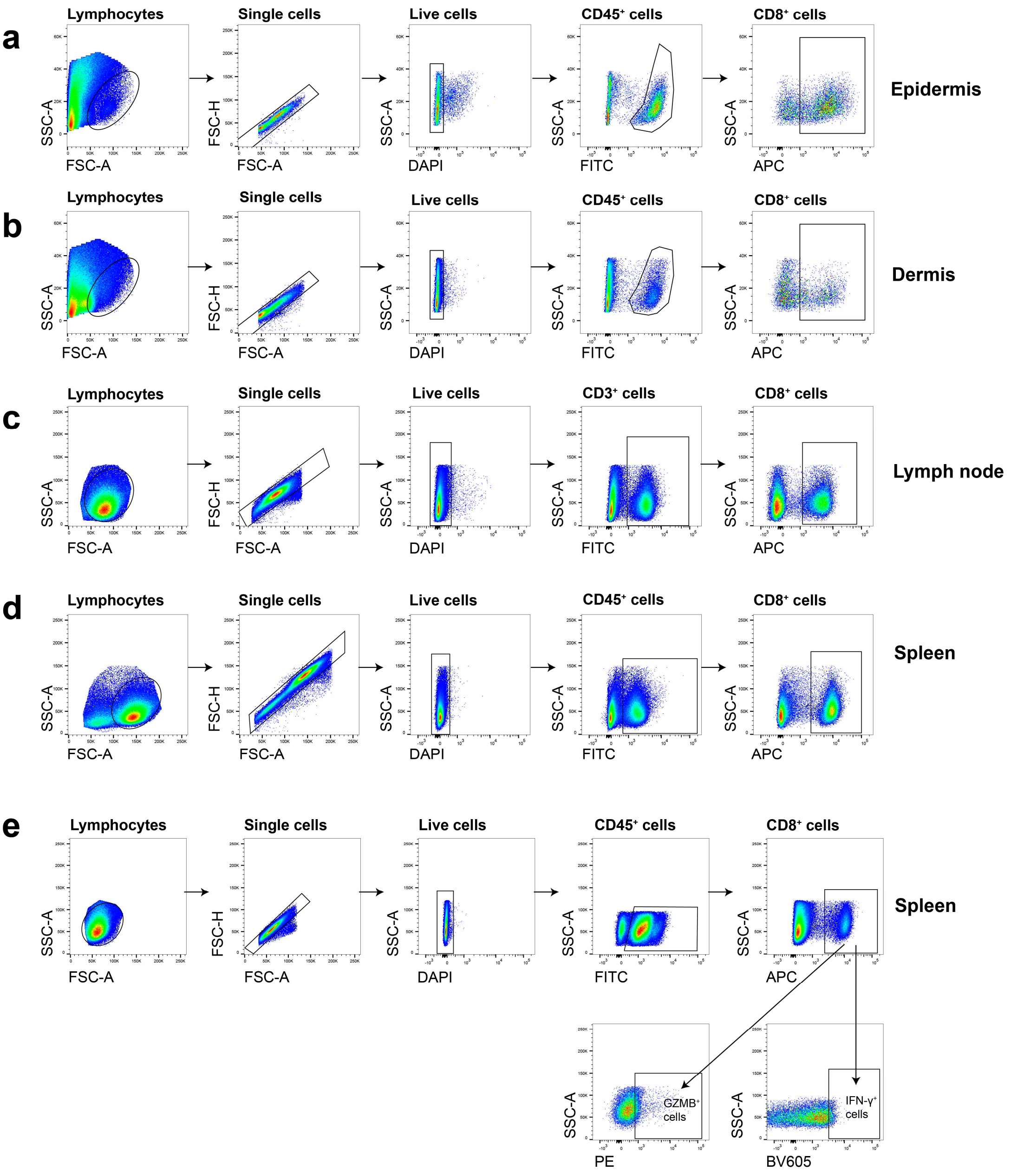

Supplementary Figure S2. Lactate promotes CD8^{+} T cell infiltration in the vitiligo mouse model.

(a) Gating strategy for flow cytometric analysis of CD8 expression in cells isolated from the epidermis of the vitiligo mouse model. (b) Gating strategy for flow cytometric analysis of CD8 expression in cells isolated from the dermis of the vitiligo mouse model. (c) Gating strategy for flow cytometric analysis of CD8 expression in cells isolated from the lymph nodes of the vitiligo mouse model. (d) Gating strategy for flow cytometric analysis of CD8

expression in cells isolated from the spleen of the vitiligo mouse model. (e) Gating strategy for flow cytometric analysis of Granzyme B and IFN-γ expression in $CD8^+$ T cells isolated from the spleen of the vitiligo mouse model.

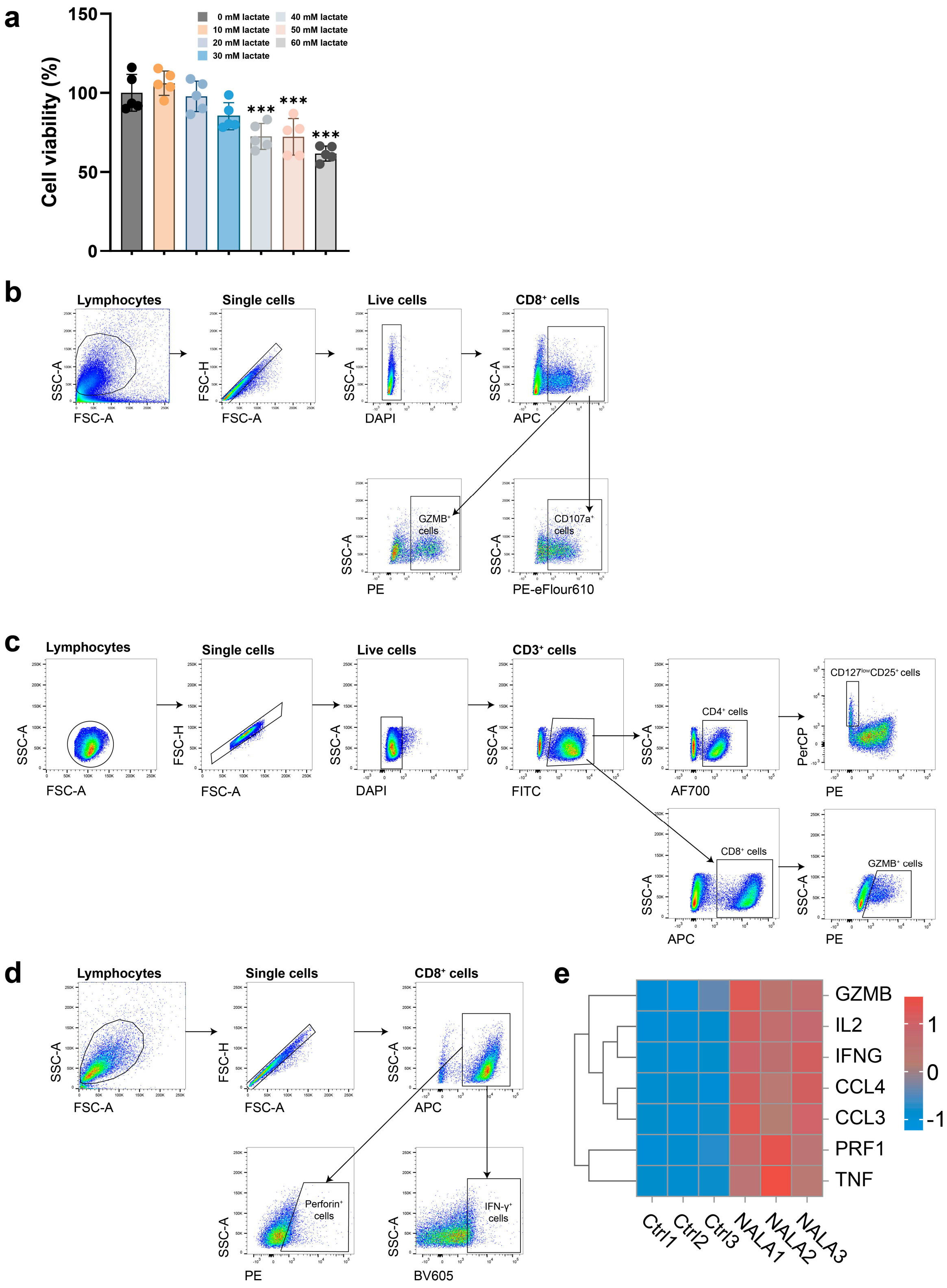

Supplementary Figure S3. Lactate enhances the cytotoxic function of CD8$^+$ T cells. (a) Viability of human CD8$^+$ T cells in response to varying concentrations of Lactate, as measured by CCK-8 assay (n = 5). (b) Gating strategy for flow cytometric analysis of Granzyme B and CD107a expression in primary human CD8$^+$ T cells. (c) Flow cytometry gating strategy for identifying Treg (CD4$^+$CD25$^+$CD127^{low}) and CD8$^+$ cell populations in human PBMCs, and the subsequent gating strategy for analyzing Granzyme B expression within the CD8$^+$ cell population. (d) Gating strategy for flow cytometric analysis of Perforin and IFN-γ expression in mouse splenic CD8$^+$ T cells. (e) Heatmap depicting the differential expression of effector function-related genes in CD8$^+$ T cells, as determined by RNA sequencing. Data are presented as mean ± SEM. ***$p < 0.001$ (one-way ANOVA with Dunnett's test for A).

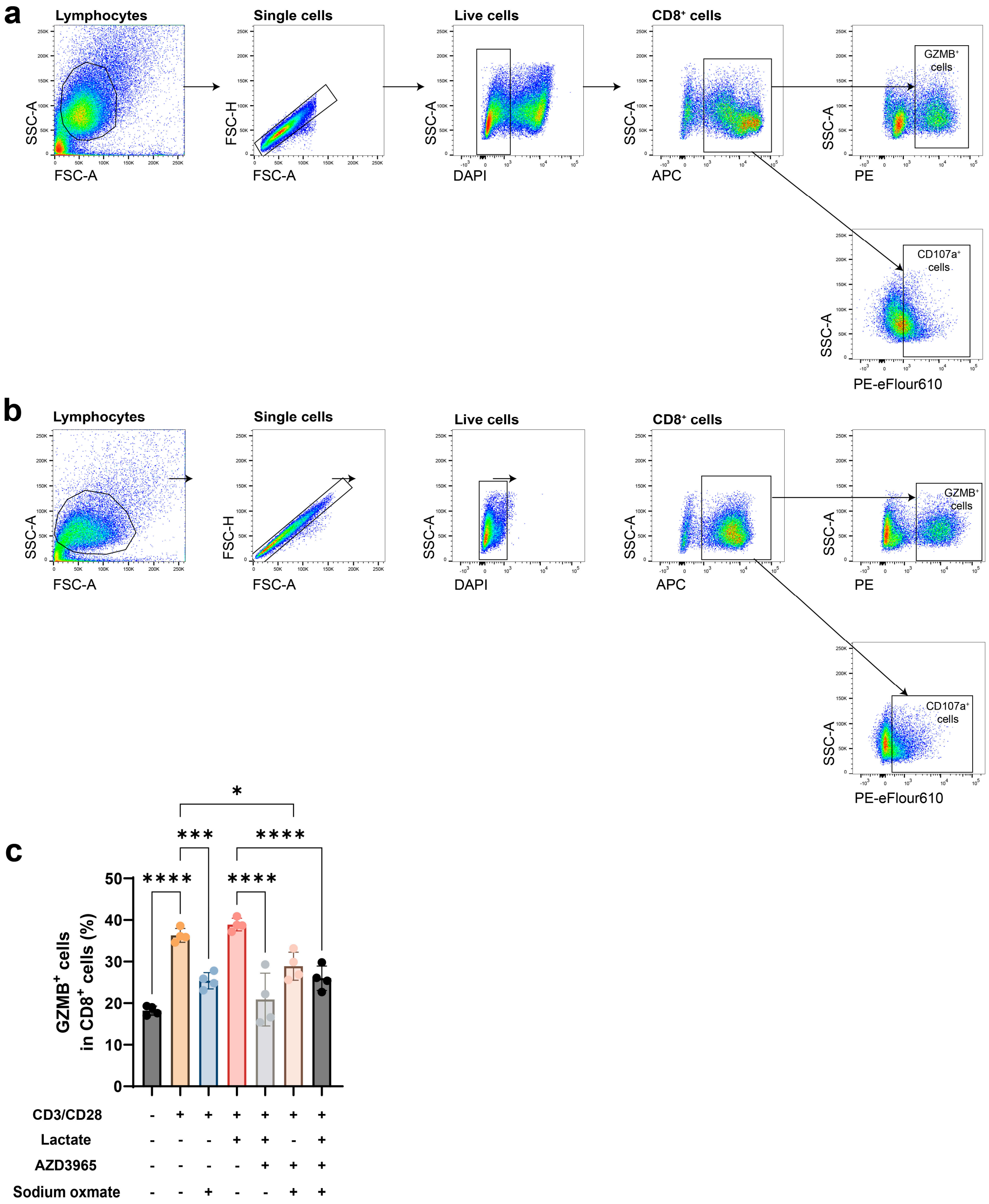

Supplementary Figure S4. Endogenous and exogenous lactate synergistically enhance CD8⁺ T cell effector function.

(a) Gating strategy for flow cytometric analysis of Granzyme B and CD107a expression in primary human CD8⁺ T cells treated with AZD3965. (b) Gating strategy for flow cytometric analysis of Granzyme B and CD107a expression in primary human CD8⁺ T cells treated with oxamate. (c) Flow cytometric analysis of the combined effects of AZD3965 and oxamate on

CD8$^+$ T cell effector function (n = 4). Data are presented as mean ± SEM. *$p < 0.05$, ***$p < 0.001$, ****$p < 0.0001$ (one-way ANOVA with Dunnett's test for C).
